# Phase 2 study of buparlisib (BKM120), a pan-class I PI3K inhibitor, in patients with metastatic triple-negative breast cancer

**DOI:** 10.1186/s13058-020-01354-y

**Published:** 2020-11-02

**Authors:** Ana C. Garrido-Castro, Cristina Saura, Romualdo Barroso-Sousa, Hao Guo, Eva Ciruelos, Begoña Bermejo, Joaquin Gavilá, Violeta Serra, Aleix Prat, Laia Paré, Pamela Céliz, Patricia Villagrasa, Yisheng Li, Jennifer Savoie, Zhan Xu, Carlos L. Arteaga, Ian E. Krop, David B. Solit, Gordon B. Mills, Lewis C. Cantley, Eric P. Winer, Nancy U. Lin, Jordi Rodon

**Affiliations:** 1 Department of Medical Oncology, Susan F. Smith Center for Women’s Cancers, Dana-Farber Cancer Institute, Harvard Medical School, 450 Brookline Avenue, Boston, MA 02215 USA; 2 Department of Medical Oncology, Vall d’Hebron University Hospital, Universitat Autònoma de Barcelona, Barcelona, Spain; 3 Vall d’Hebron Institute of Oncology, VHIO, Barcelona, Spain; 4 SOLTI Breast Cancer Research Group, Barcelona, Spain; 5 Present Address: Hospital Sírio-Libanês, Brasilia, Brazil; 6 Division of Biostatistics, Department of Data Sciences, Dana-Farber Cancer Institute, Boston, MA USA; 7 Hospital 12 de Octubre, Madrid, Spain; 8 Clinic University Hospital, INCLIVA Biomedical Research Institute, CIBERONC-ISCIII, Valencia, Spain; 9 Fundación Instituto Valenciano De Oncología, Valencia, Spain; 10 Experimental Therapeutics Group, Vall d’Hebron Institute of Oncology, Barcelona, Spain; 11 Department of Medical Oncology, Translational Genomics and Targeted Therapeutics in Solid Tumors, IDIBAPS, Hospital Clínic of Barcelona, Barcelona, Spain; 12 Department of Biostatistics, Division of Basic Sciences, The University of Texas MD Anderson Cancer Center, Houston, TX USA; 13 School of Communication, Northern Arizona University, Flagstaff, AZ USA; 14 Harold C. Simmons Comprehensive Cancer Center, UT Southwestern Medical Center, Dallas, TX USA; 15 Kravis Center for Molecular Oncology, Memorial Sloan Kettering Cancer Center, New York, NY USA; 16 Knight Cancer Institute, Oregon Health & Science University, Portland, OR USA; 17 Present Address: Division of Basic Science Research, Department of Systems Biology, The University of Texas MD Anderson Cancer Center, Houston, TX USA; 18 Sandra and Edward Meyer Cancer Center, Weill Cornell Medical College, New York, NY USA; 19 Present Address: Department of Investigational Cancer Therapeutics, The University of Texas MD Anderson Cancer Center, Houston, TX USA

**Keywords:** Buparlisib, BKM120, Triple-negative breast cancer, PI3K pathway, Phase 1

## Abstract

**Background:**

Treatment options for triple-negative breast cancer remain limited. Activation of the PI3K pathway via loss of *PTEN* and/or *INPP4B* is common. Buparlisib is an orally bioavailable, pan-class I PI3K inhibitor. We evaluated the safety and efficacy of buparlisib in patients with metastatic triple-negative breast cancer.

**Methods:**

This was a single-arm phase 2 study enrolling patients with triple-negative metastatic breast cancer. Patients were treated with buparlisib at a starting dose of 100 mg daily. The primary endpoint was clinical benefit, defined as confirmed complete response (CR), partial response (PR), or stable disease (SD) for ≥ 4 months, per RECIST 1.1. Secondary endpoints included progression-free survival (PFS), overall survival (OS), and toxicity. A subset of patients underwent pre- and on-treatment tumor tissue biopsies for correlative studies.

**Results:**

Fifty patients were enrolled. Median number of cycles was 2 (range 1–10). The clinical benefit rate was 12% (6 patients, all SD ≥ 4 months). Median PFS was 1.8 months (95% confidence interval [CI] 1.6–2.3). Median OS was 11.2 months (95% CI 6.2–25). The most frequent adverse events were fatigue (58% all grades, 8% grade 3), nausea (34% all grades, none grade 3), hyperglycemia (34% all grades, 4% grade 3), and anorexia (30% all grades, 2% grade 3). Eighteen percent of patients experienced depression (12% grade 1, 6% grade 2) and anxiety (10% grade 1, 8% grade 2). Alterations in *PIK3CA*/*AKT1*/*PTEN* were present in 6/27 patients with available targeted DNA sequencing (MSK-IMPACT), 3 of whom achieved SD as best overall response though none with clinical benefit ≥ 4 months. Of five patients with paired baseline and on-treatment biopsies, reverse phase protein arrays (RPPA) analysis demonstrated reduction of S6 phosphorylation in 2 of 3 patients who achieved SD, and in none of the patients with progressive disease.

**Conclusions:**

Buparlisib was associated with prolonged SD in a very small subset of patients with triple-negative breast cancer; however, no confirmed objective responses were observed. Downmodulation of key nodes in the PI3K pathway was observed in patients who achieved SD. PI3K pathway inhibition alone may be insufficient as a therapeutic strategy for triple-negative breast cancer.

**Trial registration:**

NCT01790932. Registered on 13 February 2013; NCT01629615. Registered on 27 June 2012.

## Introduction

Triple-negative breast cancer (estrogen receptor [ER]-negative, progesterone receptor [PgR]-negative, and human epidermal growth factor receptor 2 [HER2]-negative) accounts for approximately 12–17% of all breast cancers and is associated with poor outcomes, with an increase in the risk of recurrence compared to other breast cancer subtypes and more frequent spread to visceral organs and the central nervous system (CNS) [1–4]. Median survival after a diagnosis of metastatic triple-negative breast cancer is only approximately 1 year with current chemotherapy regimens [3, 5]. At the time this study was designed, cytotoxic chemotherapy was the mainstay of treatment, and no targeted or immunotherapeutic agents had yet gained an indication in this setting.

The phosphatidylinositol-3-kinase (PI3K) pathway is involved in diverse cellular functions, including cell growth and metabolism, migration, proliferation, and survival [6]. The molecular alterations leading to activation of the PI3K pathway in cancer are varied and include mutations in the genes encoding the PI3K alpha (*PIK3CA*) and beta (*PIK3CB*) catalytic subunits, *AKT1* mutations, and loss of expression of the phosphatidylinositol-3,4,5 trisphosphate (PIP_3_) phosphatases *PTEN* and *INPP4B.* Mutations in *PIK3CA* and *AKT1* are relatively uncommon in triple-negative breast cancer [7]; however, loss of *PTEN* and/or *INPP4B* has been reported in 30–60% of basal-like tumors [7–9].

Buparlisib (BKM120, Novartis) is an orally bioavailable, potent, pan-class I PI3K inhibitor. In the phase 1 dose-escalation study enrolling patients with advanced solid tumors, 100 mg daily was identified as the maximum tolerated dose, and one confirmed partial response was observed in a patient with triple-negative breast cancer [10, 11]. Dose-limiting toxicities were mood alteration, rash, and hyperglycemia.

The primary objective of this phase 2 study was to evaluate the clinical activity of buparlisib monotherapy in patients with metastatic triple-negative breast cancer. Secondary objectives included progression-free survival (PFS), overall survival (OS), and further characterization of the toxicity profile. A subset of patients underwent pre- and on-treatment biopsies for correlative studies. If sufficient clinical activity was observed in the first stage of the study, planned biomarker analyses to define potential expansion cohorts were to be performed in order to explore activity in a potential biomarker-selected patient population.

## Patients and methods

### Patients

The study enrolled patients with metastatic breast cancer who met the following key criteria: pathologically confirmed invasive breast cancer, ER- and PgR-negative (defined as < 1% staining by immunohistochemistry [IHC]) [12], and HER2-negative by IHC or FISH/CISH per local assessment [13]. If a patient had more than one histological result, the most recent one was considered for inclusion. Eligibility criteria included age 18 years or older; Eastern Cooperative Oncology Group performance status 0–2; at least one measurable lesion according to RECIST 1.1; adequate bone marrow, hepatic, and renal function; and fasting plasma glucose ≤ 140 mg/dL. Availability of an archival tumor specimen was also required from primary or metastatic disease. Key exclusion criteria were previous treatment with any PI3K inhibitor; symptomatic CNS metastases; concurrent use of moderate to strong modifiers of CYP3A4; or pre-existing significant mood disorder, including active major depressive episode, bipolar disorder, schizophrenia; Common Terminology Criteria for Adverse Events (CTCAE) grade ≥ 3 anxiety, or a history of suicide attempt or homicidal ideation. When the trial first opened, there was no limit on the number of prior lines of chemotherapy for metastatic breast cancer. After 30 patients were enrolled, the trial was amended to restrict the number of prior lines to 0–2 to include a less heavily pretreated population.

### Study design

This was a single-arm, phase 2 study. Two parallel protocols enrolled patients in the USA (Dana-Farber Cancer Institute, Boston, MA) or Spain (coordinating center, Vall d’Hebron, Barcelona) with a pre-specified combined analysis plan. All patients received buparlisib at a starting dose of 100 mg once daily. The planned duration of one cycle of therapy was 4 weeks. Buparlisib dose modifications were per protocol according to a pre-defined algorithm. Up to two dose reductions were allowed (to 80 mg daily and 60 mg daily). Safety assessments, including fasting plasma glucose, were conducted every 2 weeks for the first 2 cycles, and then every 4 weeks. Mood alterations were further assessed using the Patient Health Questionnaire (PHQ)-9 and Generalized Anxiety Disorder Scale (GAD)-7 on the same schedule [14, 15]. Adverse events were assessed according to the National Cancer Institute CTCAE version 4.0. Tumor assessments occurred every 2 cycles. Confirmation of response at least 4 weeks later was required to deem a confirmed complete response (CR) or partial response (PR). Paired tumor biopsies at baseline and end of cycle 1 were requested in patients with accessible disease. A minimum of 10 patients with paired biopsies enrolled within the first stage (prior to the interim analysis) was targeted. Patients were treated until disease progression, unacceptable toxicity, or withdrawal of consent.

This trial was registered with the US National Institutes of Health (ClinicalTrials.gov identifier: NCT01790932, NCT01629615). All patients provided written informed consent before the initiation of any study-related procedures or treatments.

### Study endpoints

The primary study endpoint was clinical benefit, defined as confirmed CR, confirmed PR or stable disease (SD) ≥ 4 months, as assessed by RECIST 1.1 [16]. Secondary endpoints included PFS, OS, and toxicity profile. PFS was defined as the time from date of enrollment until date of disease progression or death of any cause, whichever occurred first. If patients were alive without disease progression at the end of the study, their PFS time was censored at the date of last radiographic tumor assessment. OS was defined as time from date of enrollment until date of death from any cause. If patients were alive at the end of the study, their OS time was censored at the date of last known alive.

### Targeted DNA sequencing data

DNA was extracted from formalin-fixed paraffin-embedded (FFPE) tumor tissue samples, either archival tumor or research tumor tissue biopsies, for targeted panel sequencing. Targeted panel next-generation sequencing was performed using MSK-IMPACT testing, following previously described methods [17], using panel version IMPACT410.

### Proteomic data

Proteomic data were generated by reverse phase protein arrays (RPPA) in 5 patients with available tissue from paired baseline and on-treatment samples. RPPA was performed in the MD Anderson Cancer Center Functional Proteomics Core Facility as previously described [18, 19]. Following sonication, samples were quantitated with BCA Assay Kit and protein levels were expressed as the mean expression values in log2. Lysates were probed with antibodies validated for RPPA that are enriched for components of the PI3K pathway. The RPPA data were median-centered and scaled to one standard deviation before performing analyses. RPPA level 4 was downloaded from the Cancer Proteome Atlas (TCPA) data portal (www.TCPAportal.org) and samples with PAM50 intrinsic subtype classification were selected. The cluster analyses were displayed using Java Treeview version 3.0. Centroid linkage hierarchical clustering was performed using Gene Cluster v3.0. Biomarker data were analyzed using R (version 3.5.1).

The PI3K RPPA proteomic signature (PI3K score) was calculated as a composite biomarker PI3K score = [pAkt + pmTOR + pGSK3 + pS6K + pS6 + p4EBP1] – [INPP4b + PTEN]^10^. v3.5.1 (http://cran.r-project.org).

### Statistical analysis

The study was designed in two parts. In part 1, up to 50 patients were to be enrolled in order to describe the efficacy of buparlisib in a molecularly unselected population. An interim analysis was planned after the first 29 patients were enrolled (first stage). Assuming a prior distribution of beta (0.5, 9.5) for the response rate (*p*), the early stopping criterion of prob (*p* < 0.05 | data) > 0.95 would lead to a stopping boundary of 0 patient with clinical benefit out of the first 29 patients. The chosen prior distribution yields a prior mean response rate of 0.05 based on a prior (hypothetical) sample size of 10 patients. If any patient experienced clinical benefit, part 1 of the study was to proceed to full accrual up to 50 patients (second stage). After 50 patients were enrolled, tumor analysis was to be conducted, with correlation to clinical benefit. If clinical benefit was observed in one or more molecular subgroups (e.g., loss of *PTEN* or *INPP4B*), then part 2 of the study would open to expand specific subgroups. Patient disposition, efficacy, and safety analyses were performed using SAS.

## Results

### Patients and treatment

Fifty women were enrolled between June 2012 and September 2014 (Table 1). Median age was 53 years (range 29–79). Forty-four (44/50, 88%) patients had received neoadjuvant or adjuvant chemotherapy. Patients received a median of 1.5 lines of prior chemotherapy for metastatic disease (range 0–7).

**Table 1:** Baseline characteristics

Characteristic	No. of patients (%)
**Age, years**	
Median (range)	53 (29–79)
**Sex**	
Female	50 (100)
Male	0 (0)
**Race**	
White	46 (92)
Black	1 (2)
Asian	1 (2)
Other	2 (4)
**Ethnicity**	
Hispanic or Latino	12 (24)
Non-Hispanic	37 (74)
Unknown	1 (2)
**ECOG PS at baseline**	
0	32 (64)
1	18 (36)
**Disease-free interval (from primary diagnosis to metastatic diagnosis)**	
De novo metastatic disease	5 (10)
≤ 2 years	25 (50)
> 2 years	20 (40)
**Sites of disease at inclusion**	
CNS	2 (4)
Lung or pleural effusion	13 (26)
Liver	5 (10)
Bone	8 (16)
Breast or chest wall	15 (30)
Lymph nodes	20 (40)
Soft tissue	2 (4)
Other	4 (8)
**Previous treatment**	
Adjuvant or neoadjuvant chemotherapy	44 (88)
Adjuvant or neoadjuvant anthracycline	36 (72)
Adjuvant or neoadjuvant taxane	42 (84)
**Lines of chemotherapy for metastatic or recurrent disease**	
Median (range)	1.5 (0–7)
None	7 (14)
1 line	18 (36)
2 lines	9 (18)
3 or more lines	16 (32)
**Prior metastatic chemotherapy**	
Anthracycline	4 (8)
Taxane	14 (28)
Platinum	18 (36)
Capecitabine	23 (46)
Eribulin	8 (16)
Other	26 (52)

*CNS* central nervous system, *ECOG PS* Eastern Cooperative Oncology Group Performance Status

Follow-up information is available through September 30, 2015, for a median follow-up time of 13.8 months. The reasons for discontinuation of protocol therapy were progressive disease (*n* = 41; 82%), unacceptable toxicity (*n* = 7; 14%), physician discretion (*n* = 1; 2%), and withdrawal of consent (*n* = 1; 2%). Toxicities leading to discontinuation were grade 2 or 3 rash (*n* = 3), persistent grade 2 or higher transaminitis (*n* = 1), grade 3 hyperglycemia (*n* = 1), grade 3 fatigue and grade 2 behavior alteration (*n* = 1), and intercurrent illness (*n* = 1). At the time of data-lock, all patients had discontinued protocol therapy, 29 patients had died, 17 patients were still alive, and 4 patients were lost to follow-up.

### Efficacy

**Fig. 1:**
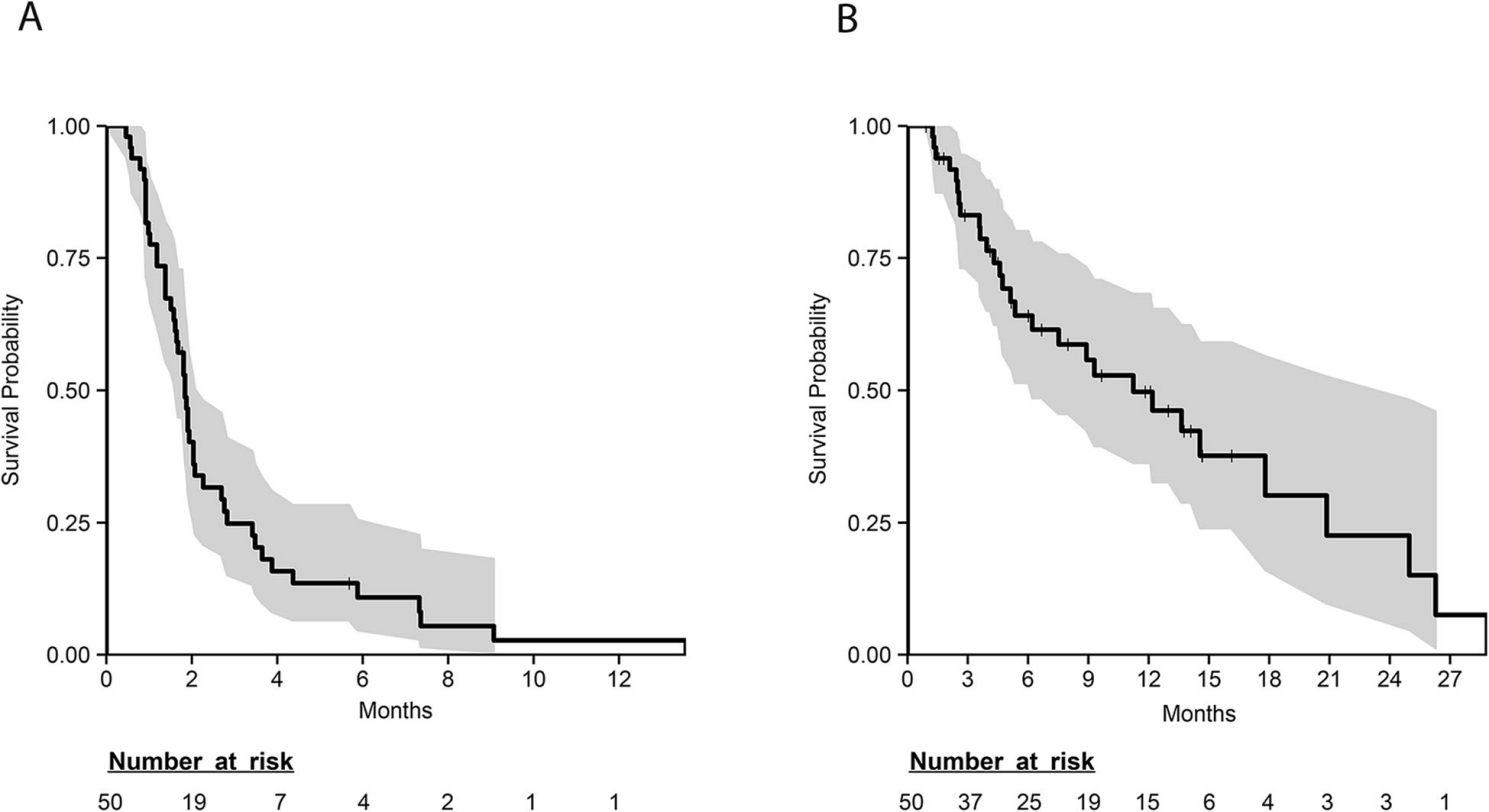
Kaplan-Meier curves for progression-free survival (**a**) and overall survival (**b**) in patients with metastatic, triple-negative breast cancer treated with buparlisib. Median PFS was 1.8 months (95% confidence interval [CI] 1.6–2.3) and median OS was 11.2 months (95% CI 6.2–25)

Of 50 patients enrolled, 37 patients were evaluable for best response, 12 patients were taken off treatment before the first restaging evaluation due to clinical progression, and one patient was taken off treatment early due to toxicity (Table 2). The first stage of accrual had a protocol-specified target accrual of 29 patients; however, one more patient was enrolled in this stage (with prior approval of a deviation) due to the protocol requirement to also enroll at least 10 patients with paired metastatic biopsies. Out of the first 30 patients, 3 experienced SD ≥ 4 months, meeting the study pre-defined primary endpoint of clinical benefit and failing to cross the early stopping boundary. Accrual thus continued toward a total of 50 patients, among which six (6/50, 12%) experienced clinical benefit of SD ≥ 4 months. One patient had an unconfirmed PR; however, there were no confirmed CR or PR. Median PFS was 1.8 months (95% CI 1.6–2.3) (Fig. 1a). Median OS was 11.2 months (95% CI 6.2–25) (Fig. 1b).

**Table 2:** Best response by RECIST 1.1

Response	No. of patients (%)
Confirmed CR	0 (0)
Confirmed PR	0 (0)
SD ≥ 4 months^§^	6 (12)
SD < 4 months	11 (22)
Progressive disease	20 (40)
Non-evaluable*	13 (26)

*CR* complete response, *PR* partial response, *SD* stable disease

^§^One patient had an unconfirmed PR

*12 patients discontinued treatment before first post-baseline assessment due to clinical progression. One patient discontinued treatment before the first post-baseline assessment due to unacceptable toxicity

### Safety

A total of 143 cycles of buparlisib were administered (Supplemental Table 1). The median number of cycles per patient was 2 (range 1–10). Dosing was held in 48% of patients; however, only 7 (14%) patients required a dose reduction. The most common reason for dose delay or hold was hepatic toxicity (6 patients).

Adverse events are summarized in Table 3. The most frequently reported adverse events (all relatedness) were fatigue (58% all grades, 8% grade 3), nausea (34% all grades, none grade 3), hyperglycemia (34% all grades, 4% grade 3), and anorexia (30% all grades, 2% grade 3). Any grade depression and anxiety were each reported in 9 patients, none of which were grade 3. QTc prolongation was noted in 13 patients (10 [20%] grade 1; 3 [6%] grade 2; no grade 3 or 4). As expected, hyperglycemia was noted, with one (2%) patient experiencing grade 2 and two (4%) patients experiencing grade 3 hyperglycemia. Three (6%) patients required a change or addition of medication for glucose control (*n* = 1, addition of metformin; *n* = 1, increase in dose of metformin, addition of glimepiride; *n* = 1, increase in dose of metformin, addition of insulin).

**Table 3:** All grade adverse events reported in ≥10% of patients or any reported grade 3 events (all relatedness), *n* = 50

AE description	Number (%) of AE by CTCAE grading			
Frequency	G1	G2	G3	Total*
**Fatigue**	15 (30)	10 (20)	4 (8)	29 (58)
**Nausea**	12 (24)	5 (10)	0 (0)	17 (34)
**Hyperglycemia**	14 (28)	1 (2)	2 (4)	17 (34)
**Anorexia**	12 (24)	2 (4)	1 (2)	15 (30)
**QTc prolongation**	10 (20)	3 (6)	0 (0)	13 (26)
**Alanine aminotransferase increased**	3 (6)	2 (4)	5 (10)	10 (20)
**Anxiety**	5 (10)	4 (8)	0 (0)	9 (18)
**Depression**	6 (12)	3 (6)	0 (0)	9 (18)
**Diarrhea**	8 (16)	1 (2)	0 (0)	9 (18)
**Aspartate aminotransferase increased**	3 (6)	1 (2)	4 (8)	8 (16)
**Psychiatric disorders—other**	4 (8)	3 (6)	0 (0)	7 (14)
**Gastrointestinal disorders—other**	5 (10)	0 (0)	1 (2)	6 (12)
**Rash acneiform**	5 (10)	0 (0)	1 (2)	6 (12)
**Constipation**	6 (12)	0 (0)	0 (0)	6 (12)
**Cough**	5 (10)	1 (2)	0 (0)	6 (12)
**Insomnia**	6 (12)	0 (0)	0 (0)	6 (12)
**Pain**	1 (2)	5 (10)	0 (0)	6 (12)
**Dyspnea**	2 (4)	3 (6)	0 (0)	5 (10)
**Mucositis oral**	4 (8)	1 (2)	0 (0)	5 (10)
**Rash maculo-papular**	0 (0)	0 (0)	2 (4)	2 (4)
**Alkalosis**	0 (0)	0 (0)	1 (2)	1 (2)
**Dry skin**	0 (0)	0 (0)	1 (2)	1 (2)
**Hepatic failure**	0 (0)	0 (0)	1 (2)	1 (2)
**Hyponatremia**	0 (0)	0 (0)	1 (2)	1 (2)
**Nervous system disorders—other**	0 (0)	0 (0)	1 (2)	1 (2)
**Pain in extremity**	0 (0)	0 (0)	1 (2)	1 (2)
**Papulopustular rash**	0 (0)	0 (0)	1 (2)	1 (2)
**Skin and subcutaneous tissue disorders—other**	0 (0)	0 (0)	1 (2)	1 (2)

*AE* adverse event, *CTCAE* Common Terminology Criteria for Adverse Events, *G* grade

*No grade 4 or 5 events were reported

Results of neuropsychiatric assessments using the PHQ-9 and GAD-7 instruments are displayed in Table 4. Completion rates of the instruments exceeded 70% at all timepoints. Moderate (grade 2) depressive symptoms were reported by 11 (22%) patients on study, and in 2 (4%) patients at baseline. Moderate anxiety symptoms were reported by 8 (16%) patients on study, in contrast to 2 (4%) patients at baseline. Three patients reported treatment-emergent grade 3 depressive symptoms, one patient reported grade 3 anxiety symptoms, and one patient reported both treatment-emergent grade 3 depressive and anxiety symptoms according to these scales. Supplemental Table 2 collates data from patients with at least one neuropsychiatric assessment by PHQ-9 or GAD-7 showing a score ≥ 10, the corresponding reported psychiatric adverse events by CTCAE 4.0 by investigator assessment, and medical management of psychiatric adverse events. Of note, standard investigator-reported CTCAE psychiatric adverse events (e.g., depression, anxiety) underestimated psychiatric symptoms as compared to patient-reported neuropsychiatric outcomes as assessed with the PHQ-9 and GAD-7 scales.

**Table 4:** Neuropsychiatric assessment

**Completion rate of PHQ-9 and GAD-7 scales by timepoint**					
**PHQ-9**	**No. patients required**			**No. patients completed (% of required)**	
Baseline	50			49 (98)	
Cycle 1 day 15	45			44 (98)	
Cycle 2 day 1	40			38 (95)	
Cycle 2 day 15	26			26 (100)	
Cycle 3 day 1	19			15 (79)	
Off treatment	50			36 (72)	
**GAD-7**	**No. patients required**			**No. patients completed (% of required)**	
Baseline	50			49 (98)	
Cycle 1 day 15	45			44 (98)	
Cycle 2 day 1	40			38 (95)	
Cycle 2 day 15	26			26 (100)	
Cycle 3 day 1	19			15 (79)	
Off treatment	50			36 (72)	
**Assessment**					
	**Overall (%)**	**Normal, n (%)**	**Grade 1, n (%)**	**Grade 2, n (%)**	**Grade 3, n (%)**
**PHQ-9**					
Baseline	49	33 (67)	14 (29)	2 (4)	0 (0)
Highest grade on study	50	11 (22)	24 (48)	11 (22)	4 (8)
**GAD-7**					
Baseline	49	33 (67)	14 (29)	2 (4)	0 (0)
Highest grade on study	50	21 (42)	19 (38)	8 (16)	2 (4)
**Change to medications for mood disorder**					
	Total (*n* = 50), n (%)				
No	- 45 (90)				
Yes	- 5 (10)				

PHQ-9 and GAD-7 Score Severity CTCAE grading: 0–4 normal; 5–9 grade 1; 10–14 grade 2; ≥ 15 grade 3

*GAD-7* Generalized Anxiety Disorder Scale, *PHQ-9* Patient Health Questionnaire-9

### Correlative studies

**Fig. 2:**
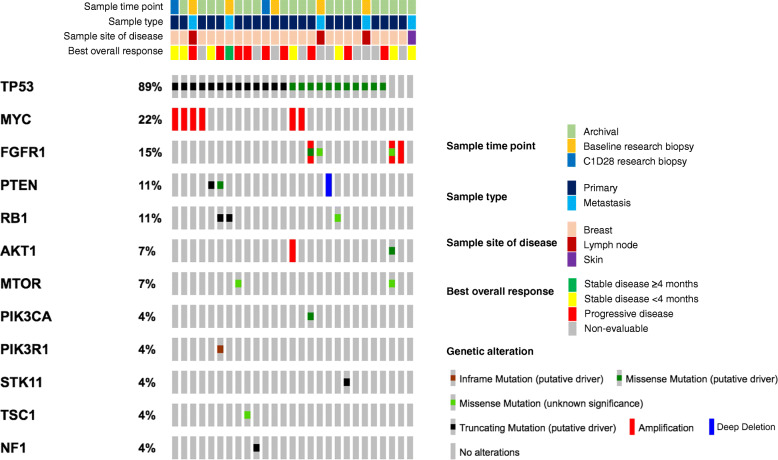
Co-mutation/CNV plot of tumor samples analyzed with targeted next-generation sequencing. Co-mutation and copy number variation plot for samples analyzed with MSK-IMPACT panel testing (*n* = 27 samples; *n* = 27 patients). Included here are genes related to PI3K pathway and drug resistance with at least one identified alteration in the cohort. Best overall response is displayed for each patient. Six samples harbored an alteration in *PIK3CA* (E542K mutation, *n* = 1), *AKT1* (E17K mutation, *n* = 1; *AKT1* amplification, *n* = 1), or *PTEN* (D24H mutation, *n* = 1; K237fs mutation, *n* = 1; *PTEN* deep deletion, *n* = 1). Alterations in *PIK3CA*, *AKT1*, or *PTEN* were observed in 3 of 8 patients (37.5%) who achieved SD, and in 2 of 9 (22.2%) patients with progressive disease as best overall response

Targeted DNA sequencing was performed using MSK-IMPACT testing on archival or research tumor tissue biopsies. Gene single-nucleotide variants (SNV) and copy number variation (CNV) results were available in tumor samples from 27 patients, including 20 archival FFPE samples and 7 research core biopsies (baseline, *n* = 5; C1D28, *n* = 2). Of these 27 samples, 6 (22.2%) harbored an alteration in *PIK3CA* (E542K mutation, *n* = 1), *AKT1* (E17K mutation, *n* = 1; *AKT1* amplification, *n* = 1), or *PTEN* (D24H mutation, *n* = 1; K237fs mutation, *n* = 1; *PTEN* deep deletion, *n* = 1). In the sample with *PTEN* deletion (log ratio − 2.87), the TP53 variant allele frequency was 0.57, supporting likely homozygous loss of PTEN. Additional alterations in genes in the PI3K pathway [20] are displayed in Fig. 2.

As specified in the “Patients and methods” section, the trial was originally designed to evaluate predictive biomarkers of response to buparlisib (with particular emphasis on alterations in the PI3K/PTEN pathway), with potential expansion cohorts enriched for the identified biomarkers. However, we did not observe sufficient clinical activity to move on to expansion cohorts or to test for potential predictors of clinical benefit. Thus, the ability to correlate genomic findings with response to buparlisib was limited. In the six patients with tumor alterations in *PIK3CA*, *AKT1*, or *PTEN*, the best overall response was SD < 4 months (*n* = 3, including the two patients with *AKT1*-altered tumors and one patient with *PTEN* K237fs mutation), progressive disease (*n* = 2), and non-evaluable (*n* = 1). Of the remaining 21 patients without identified alterations in *PIK3CA*/*AKT1*/*PTEN*, the best overall response was SD ≥ 4 months (*n* = 1), SD < 4 months (*n* = 4), progressive disease (*n* = 7), and non-evaluable (*n* = 9). In the single patient who achieved clinical benefit ≥ 4 months from buparlisib with available SNV/CNV results, splicing mutations in *TP53* and *RB1* were identified in the baseline metastatic research biopsy.

**Fig. 3:**
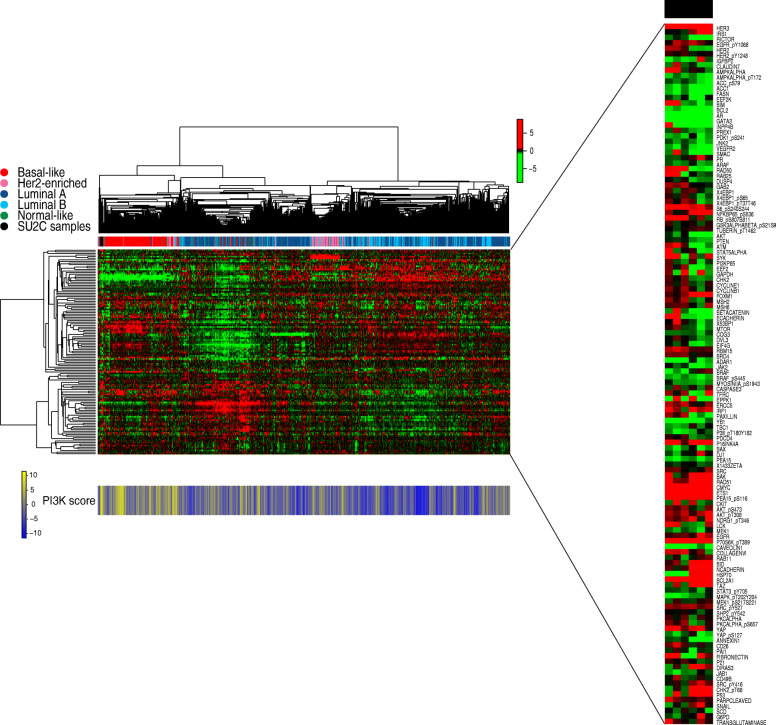
Unsupervised clustering and PI3K signature score based on the RBN dataset. Heatmap depicting protein levels after unsupervised hierarchical clustering of 826 TCGA samples plus 6 baseline SU2C samples and 128 antibodies. Protein levels are indicated on a low-to-high scale (green-black-red). Annotation bars include the PAM50 intrinsic subtype classification. The PI3K score is shown in the lower panel. SU2C samples (black) are located in close proximity to triple-negative breast cancer samples from TCGA (red)

**Fig. 4:**
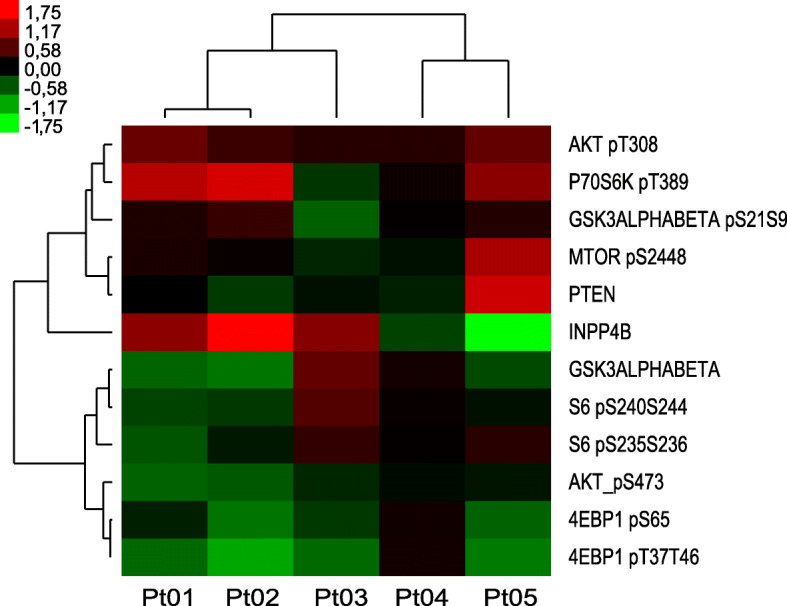
Unsupervised clustering based on the delta values of the paired samples. Heatmap depicting the delta values in the paired samples of the PI3K signature-related proteins ordered from upstream (top) to downstream (bottom). Protein levels are indicated on a low-to-high scale (green-black-red)

Seventeen patients underwent a baseline biopsy, 6 of them had sufficient tissue for proteomic analysis and 5 patients had available paired baseline and on-treatment biopsies. Baseline samples (*n* = 6) were analyzed by RPPA and compared with the TCGA dataset (Fig. 3, SU2C samples in black are located in close proximity to triple-negative breast cancer samples from TCGA in red). All samples from this study clustered among the TCGA triple-negative breast tumors. As shown in Fig. 3, five out of six exhibited a PI3K score consistent with PI3K pathway activation. We further analyzed the PI3K pathway downmodulation after treatment with buparlisib in the five patients exhibiting an activated PI3K signature in whom a paired on-treatment biopsy was also available (Fig. 4). Integral downmodulation of key nodes in the pathway including AKT S473, S6 S235/S236, S6 S240/S244, 4EBP1 S65, and 4EBP1 T37/T46 was exclusively observed in two paired patient samples (Pt01, Pt02), albeit there was evidence of concomitant feedback activation on AKT T308 and p70S6K T389. With regards to treatment response, these two patients experienced disease stabilization, along with Pt04, for whom there was no pathway modulation observed. Pt03 and Pt05 experienced progressive disease as the best overall response and their tumor samples showed no change in S6 phosphorylation. As previously suggested by Elkabets et al. for the PI3Kα inhibitor alpelisib, reduction of S6 phosphorylation appeared to be a key component of pathway modulation associated to treatment response [21].

## Discussion

Given the observation that the majority of triple-negative breast tumors demonstrate activation of the PI3K pathway, we tested the pan-class I PI3K inhibitor buparlisib in this phase 2 trial. In a small subset of patients in whom paired baseline and on-treatment tumor biopsies were available, we observed evidence of target inhibition in 2 of 3 patients with SD as best response, and none of 2 patients with progressive disease as best response. Overall, only 12% of patients achieved prolonged SD, and we did not observe any confirmed objective responses. In those with available targeted panel DNA sequencing results, genomic alterations in *PIK3CA*, *AKT1*, or *PTEN* were present in 3 of 8 (37.5%) patients who achieved SD, and in 2 of 9 (22.2%) patients with progressive disease as best overall response. Median PFS for the overall study population was less than 2 months. Given the lack of a strong clinical signal, we chose not to open expansion cohorts in molecularly selected subsets of triple-negative breast cancer.

Other trials have demonstrated that treatment with single-agent inhibitors of the PI3K pathway has only limited efficacy in patients with metastatic breast cancer, whether unselected [10] or selected for the presence of *PIK3CA/AKT/PTEN* alterations in the tumor [22]. Our study adds to the previous reports by demonstrating that buparlisib has essentially no single-agent activity in unselected triple-negative breast cancer, and the development of buparlisib has been discontinued in breast cancer. The toxicity profile we observed is in keeping with other reports of buparlisib at the studied dose and schedule. Notably, any grade anxiety and any grade depression were reported in 18% of patients. Buparlisib is one of the PI3K inhibitors with highest blood-brain barrier penetration [23]. However, the physiopathology of mood disorders associated with pan-PI3K inhibitors has not been well characterized, and it remains unclear whether the higher incidence of neuropsychiatric effects observed with buparlisib compared to isoform-selective PI3K inhibitors is due to its elevated CNS penetration or to other pharmacokinetic differences. Pan-PI3K inhibitors have broader off-target effects, in addition to on-target inhibition of each of the class I PI3K catalytic isoforms, thus resulting in a less favorable toxicity profile compared to selective p110-alpha inhibitors [24].

Of interest is the role of combining PI3K inhibitors with other therapeutic modalities. Based on preclinical data suggesting that adding a PI3K inhibitor to endocrine therapy may overcome resistance, two large phase 3 clinical trials, BELLE-2 [25] and BELLE-3 [26], evaluated the efficacy of buparlisib plus fulvestrant versus placebo plus fulvestrant in postmenopausal patients with ER-positive breast cancer refractory to aromatase inhibitors. In addition, BELLE-3 required patients to be resistant to mTOR inhibitors. Both trials met their primary endpoint in the full population with the combination leading to modest increases in PFS. Safety data were consistent with that previously seen with buparlisib-based regimens, with transaminase elevation and hyperglycemia as the most common grade 3–4 adverse events. Among patients with ER-positive breast cancer whose tumors harbor an activating *PIK3CA* mutation, results of the SOLAR-1 trial demonstrated a clear PFS improvement with the alpha-specific PI3K inhibitor, alpelisib, in combination with fulvestrant [27]. Similarly, in the SANDPIPER trial, patients with *PIK3CA*-mutant tumors reportedly had increased median PFS with the addition of taselisib (mutant-selective PI3K inhibitor) to fulvestrant [28].

Other studies have evaluated combinatorial approaches of PI3K pathway inhibition with chemotherapy. In a phase 1 dose escalation study of buparlisib combined with capecitabine in patients with metastatic breast cancer (*n* = 25), one patient with triple-negative breast cancer treated at the maximum-tolerated dose (buparlisib 100 mg daily; capecitabine 1000 mg/m^2^ twice daily) achieved a CR after receipt of 11 cycles of therapy, and ultimately discontinued treatment after 32 cycles due to mood changes [29]. Genomic testing confirmed that the tumor was of basal-like intrinsic subtype, without evidence of an activating *PIK3CA* mutation. Four additional patients (two with ER-positive, one with HER2-positive, and one with triple-negative breast cancer) exhibited a PR to treatment, and the remaining 12 evaluable patients experienced SD, suggesting potential clinical activity. In contrast, the randomized phase 2 trial comparing buparlisib plus paclitaxel versus placebo plus paclitaxel in patients with HER2-negative breast cancer was stopped early for futility, as the addition of the PI3K inhibitor failed to demonstrate a significant PFS improvement in either the overall study population or in the prespecified cohort of patients with PI3K-activated tumors [30]. In the hormone receptor-negative population enrolled on the study (99/416 patients, 24%), median PFS was numerically inferior with buparlisib compared to placebo (5.5 vs. 9.3 months, respectively). Notably, ipatasertib, a selective AKT inhibitor, improved PFS in combination with paclitaxel (compared to placebo plus paclitaxel) as first-line therapy for metastatic triple-negative breast cancer in the LOTUS phase 2 trial [31]. This benefit was observed in both the intent-to-treat population (6.2 vs. 4.9 months, hazard ratio [HR] 0.60) and in patients with *PIK3CA*/*AKT*/*PTEN*-altered tumors (9.0 vs. 4.9 months, HR 0.44), leading to the design of the ongoing randomized phase 3 trial exploring the combination in preselected triple-negative breast cancer with activation of the PI3K pathway (NCT03337724). The addition of capivasertib, another selective AKT inhibitor, to paclitaxel also significantly increased PFS, with a trend toward longer OS, in patients with prespecified alterations in *PI3KCA*, *AKT*, or *PTEN*; however, this benefit was not seen in patients without aberrant pathway changes [32]. Confirmation of activity will be explored in an ongoing randomized phase 3 study which will enroll patients with untreated locally advanced or metastatic triple-negative breast cancer (NCT03997123). Similarly, the alpha-specific PI3K inhibitor alpelisib showed promising activity in combination with albumin-bound paclitaxel in patients with metastatic HER2-negative breast cancer, most (30/43, 70%) of whom had received at most one prior line of chemotherapy in the metastatic setting [33]. In the triple-negative breast cancer subgroup with available response data, unselected for PI3K status, the objective response rate was 58% (7/12). In the overall study population, numerically greater objective response rate (74% vs. 48%), clinical benefit rate (100% vs. 61%), and median PFS (13 vs. 7 months, HR 0.40) were observed among patients with PI3K pathway-activation compared to those without *PIK3CA*-activating or *PTEN*-inactivating mutations, leading to the design of an ongoing phase 3 randomized study of alpelisib or placebo plus albumin-bound paclitaxel as first- or second-line therapy for patients with *PIK3CA*-mutant or PTEN loss (without *PIK3CA* mutation) advanced triple-negative breast cancer (NCT04251533).

It is unclear whether the lack of effectiveness of treatment with single-agent pan-PI3K inhibitors in triple-negative breast cancer is related to adaptive activation of compensatory signaling pathways or due to a failure to achieve sufficient target inhibition and PI3K pathway suppression due to toxicity. PTEN loss has been found to lead to a convergent PTEN-null phenotype resistant to PI3K-alpha inhibition [34]. Given that 60% of triple-negative breast cancers harbor PTEN genomic alterations [7, 8], it would be important to define whether such alterations could also be responsible for de novo resistance to PI3K inhibitors as well. Furthermore, given the variable efficacy of targeting the PI3K pathway to date in unselected or PI3K-activated triple-negative breast cancer, additional biomarker analyses are needed to better understand the putative oncogenic role of these genes in this breast cancer subtype.

Our study had several limitations. First, we tested only single-agent buparlisib and cannot rule out that combination therapies might provide additional efficacy. Since we initiated this study, the treatment landscape for patients with metastatic triple-negative breast cancer has evolved considerably, and two targeted approaches were recently approved: the anti-PD-L1 inhibitor atezolizumab in combination with *nab*-paclitaxel in PD-L1-positive triple-negative breast cancer [35], and the PARP inhibitors olaparib or talazoparib for germline *BRCA1*/*BRCA2* mutation carriers [36, 37]. There are now preliminary data supporting the molecular rationale to test combinations of PI3K inhibitors with these agents [38]. For example, based on a strong preclinical synergistic signal combining buparlisib and olaparib [39], a phase 1 clinical trial evaluating the combination was launched in advanced triple-negative breast cancer and ovarian carcinoma [40]. Results of the study demonstrated feasibility and promising anti-cancer activity for this combination, though transaminase elevations and neuropsychiatric adverse events were common and responsible for dose-limiting toxicities. There are also emerging data that PTEN loss is associated with resistance to immunotherapy, raising the question of whether combinations of inhibitors of PI3K/AKT/mTOR signaling with immune checkpoint inhibitors could provide additional clinical efficacy [41]. Second, although we had originally designed the study to evaluate predictive biomarkers of response to buparlisib, with potential expansion cohorts enriched for the identified biomarkers, we did not observe sufficient clinical activity to move on to expansion cohorts or to test for potential predictors of clinical benefit. In addition, we could not establish if the degree of PI3K downmodulation was sufficient to be consistent with antitumor activity.

## Conclusions

In summary, buparlisib, when given as a single agent in patients with triple-negative breast cancer, was not associated with a strong clinical signal of efficacy, though a small subset of patients did achieve prolonged SD. Downmodulation of key nodes in the PI3K pathway was observed in patients who achieved tumor stabilization. The toxicity profile of buparlisib was consistent with previous reports, highlighting the pharmacological limitations of pan-PI3K inhibition compared to selective PI3K isoform inhibitors that may achieve improved efficacy with fewer side effects. Although our results do not support additional testing of single-agent PI3K inhibitors in triple-negative breast cancer, they do not preclude the potential benefit for the efficacy of rational combinations including selective PI3K pathway inhibitors.

## Supplementary information

**Additional file 1.**

## Data Availability

The datasets used and/or analyzed during the current study are available from the corresponding author on reasonable request.

## Abbreviations

CNS: Central nervous system
CR: Complete response
CTCAE: Common Terminology Criteria for Adverse Events
CNV: Copy number variation
ER: Estrogen receptor
FFPE: Formalin-fixed paraffin-embedded
GAD: Generalized Anxiety Disorder Scale
HER2: Human epidermal growth factor receptor 2
HR: Hazard ratio
IHC: Immunohistochemistry
OS: Overall survival
PFS: Progression-free survival
PgR: Progesterone receptor
PHQ: Patient Health Questionnaire
PI3K: Phosphatidylinositol-3-kinase
PR: Partial response
RPPA: Reverse phase protein arrays
SD: Stable disease
SNV: Single-nucleotide variants
TCPA: The Cancer Proteome Atlas
