# Supplementary material for: Phase 2 study of buparlisib (BKM120), a pan-class I PI3K inhibitor, in patients with metastatic triple-negative breast cancer
Source: Breast Cancer Res. 2020 Nov 2;22:120. doi: 10.1186/s13058-020-01354-y (PMC7607628; doi:10.1186/s13058-020-01354-y)
Supplement: Supplementary file 1 — Additional file 1. [file 13058_2020_1354_MOESM1_ESM.docx]

**Phase 2 Study of Buparlisib (BKM120), a pan-class I PI3K inhibitor, in Patients with Metastatic, Triple-Negative Breast Cancer**

Ana C. Garrido-Castro^*^, Cristina Saura^*^, Romualdo Barroso de Sousa, Hao Guo, Eva Ciruelos, Begoña Bermejo, Joaquin Gavilá, Violeta Serra, Aleix Prat, Laia Paré, Pamela Céliz, Patricia Villagrasa, Yisheng Li, Jennifer Savoie, Zhan Xu, Carlos L. Arteaga, Ian E. Krop, David B. Solit, Gordon B. Mills^*^, Lewis C. Cantley, Eric P. Winer, Nancy U. Lin^**^, Jordi Rodon^**^

*these authors contributed equally

**co-senior authors

**Corresponding Author:**

Nancy U. Lin, MD

Susan F. Smith Center for Women’s Cancers

Dana-Farber Cancer Institute

450 Brookline Avenue

Boston, MA 02215

Tel: 617-632-3800

Fax: 617-632-1930

Email: [nlin@partners.org](mailto:nlin@partners.org)

**Supplemental Tables: 2**

**Supplemental Table 1. Treatment Details**

| **Treatment Details** | **N** |
| --- | --- |
| **Total number of cycles administered** | 143 |
| **Median number of cycles per patient (range)** | 2 (1-10) |
| **Number of patients with dose delay or hold** | 24 (48%) |
| **Number of patients with dose reduction** | 7 (14%) |
| **Reasons for dose delay or hold** |  |
| **Toxicity** | 20 |
| Hepatic | 6 |
| Skin | 4 |
| Neurotoxicity | 2 |
| Hyperglycemia | 1 |
| Dyspepsia | 1 |
| Fatigue | 2 |
| Hematologic | 1 |
| Fatigue, nausea, anorexia | 1 |
| Hospitalization for pain due to humerus fracture | 1 |
| Shingles | 1 |
| **Scheduling problems** | 1 |
| **Others** | 3 |
| **Reasons for dose reduction** |  |
| **Toxicity** | 7 |
| Hepatic | 2 |
| Fatigue | 1 |
| Gastrointestinal | 1 |
| Rash | 1 |
| Hyperglycemia | 1 |
| Mood alteration | 1 |

**Supplemental Table 2. Patients with neuropsychiatric assessments (NPA) by PHQ-9 or GAD-7 scored ≥10, corresponding reported psychiatric adverse events by CTCAE 4.0, and medical management**

| **Patient** | **Timepoint** | **PHQ-9 score** | **GAD-7 score** | **Psychiatric AE by CTCAE v4.0** | **Management of toxicity by dose modification** | **Psychiatric medication received** |
| --- | --- | --- | --- | --- | --- | --- |
| 1 | Cycle 5 Day 1 | 7 | 10 | N.R. | No | No |
| 2 | Cycle 1 Day 15 | 20 | 10 | Grade 2 mood alteration; Grade 1 anxiety | No | No |
| 3 | Baseline | 12 | 8 | Grade 1 depression; grade 1 anxiety | No | No |
|  | Cycle 1 Day 15 | 7 | 10 |  |  |  |
| 4 | Off treatment (Cycle 1) | 10 | 2 | N.R. | No | No |
| 5 | Cycle 2 Day 1 | 11 | 3 | N.R. | No | No |
| 6 | Cycle 1 Day 15 | 7 | 13 | Psychiatric disorder, other: GAD-7 results | No | No |
| 7 | Cycle 2 Day 1 | 10 | 11 | N.R. | No | No |
| 8 | Cycle 1 Day 15 | 17 | 8 | Grade 2 depression; grade 1 anxiety | Dose held around the time of psychiatric AE | Yes |
|  | Cycle 2 Day 1 | 11 | 9 |  |  |  |
| 9 | Baseline | 6 | 12 | Grade 2 depression; grade 2 anxiety; grade 2 insomnia | No | No |
|  | Cycle 1 Day 15 | 12 | 11 |  |  |  |
|  | Off treatment (Cycle 2) | 6 | 10 |  |  |  |
| 10 | Cycle 2 Day 1 | 11 | 5 | N.R. | No | No |
| 11 | Off treatment  (Cycle 3) | 14 | 18 | N.R. | No | No |
| 12 | Baseline | 11 | 2 | N.R. | No | No |
|  | Cycle 3 Day 1 | 13 | 4 |  |  |  |
| 13 | Cycle 2 Day 1 | 17 | 8 | Grade 1 anxiety | No | No |
| 14 | Cycle 1 Day 15 | 7 | 13 | Grade 2 depression; grade 2 anxiety | No | No |
|  | Cycle 2 Day 15 | 13 | 9 |  |  |  |
| 15 | Cycle 1 Day 15 | 10 | 6 | N.R. | No | Yes |
|  | Off treatment  (Cycle 1) | 13 | 6 |  |  |  |
| 16 | Cycle 1 Day 15 | 12 | 7 | Grade 1 depression | No | Yes |
|  | Cycle 2 Day 15 | 14 | 0 |  |  |  |
| 17 | Baseline | 3 | 11 | N.R. | No | No |
| 18 | Cycle 1 Day 8 | 21 | 17 | N.R. | Dose reduced | Yes |
| 19 | No PHQ-9 ≥10 or GAD-7 ≥ 10 | | | Grade 2 anxiety | No | Yes |

AE: adverse event; CTCAE: Common Terminology Criteria for Adverse Events; GAD-7: Generalized Anxiety Disorder Scale-7; N.R.: not reported; PHQ-9: Patient Health Questionnaire-9.

PHQ-9 and GAD-7 Score Severity CTCAE grading: 0-4 Normal; 5-9 Grade 1; 10-14 Grade 2; ≥ 15 Grade 3.
